# Identification of Retinal Transformation Hot Spots in Developing *Drosophila* Epithelia

**DOI:** 10.1371/journal.pone.0008510

**Published:** 2010-01-07

**Authors:** Claire L. Salzer, Justin P. Kumar

**Affiliations:** Department of Biology, Indiana University, Bloomington, Indiana, United States of America; University of Texas MD Anderson Cancer Center, United States of America

## Abstract

**Background:**

The retinal determination (RD) network is an evolutionarily conserved regulatory circuit that governs early events in the development of eyes throughout the animal kingdom. Ectopic expression of many members of this network leads to the transformation of non-retinal epithelia into eye tissue. An often-overlooked observation is that only particular cell-populations within a handful of tissues are capable of having their primary developmental instructions superseded and overruled.

**Methodology/Preliminary Findings:**

Here we confirm that indeed, only a discrete number of cell populations within the imaginal discs that give rise to the head, antenna, legs, wings and halteres have the cellular plasticity to have their developmental fates altered. In contrast to previous reports, we find that all transformable cell populations do not lie within the TGFβ or Hedgehog signaling domains. Additionally neither signaling cascade alone is sufficient for non-retinal cell types to be converted into retinal tissue. The transformation “hot spots” that we have identified appear to coincide with several previously defined transdetermination “weak spots”, suggesting that ectopic eye formation is less the result of one network overriding the orders of another, as previously thought, but rather is the physical manifestation of redirecting cell populations of enormous cellular plasticity. We also demonstrate that the initiation of eye formation in non-retinal tissues occurs asynchronously compared to that of the normal eye suggesting that retinal development is not under the control of a global developmental clock.

**Conclusions/Significance:**

We conclude that the subregions of non-retinal tissues that are capable of supporting eye formation represent specialized cell-populations that have a different level of plasticity than other cells within these tissues and may be the founder cells of each tissue.

## Introduction

The retinal determination (RD) network is an evolutionarily conserved regulatory circuit that governs early events in the development of eyes throughout the animal kingdom. In *Drosophila*, removal of individual RD genes leads to an inhibition of eye formation while forced expression of these genes is sufficient to redirect the fate of non-retinal tissues [1]. The *Pax6/eyeless* (*ey*) gene exemplifies these characteristics as *ey* mutants have retinal defects that range from partial to complete loss of eye tissue. In contrast, ectopic expression induces eye formation in the imaginal discs that give rise to the developing antenna, legs, wings and halteres [2], [3]. Scattered evidence from the literature has suggested that the ability to induce eye formation is not unlimited [2], [4]–[13]. However, a complete understanding of the mechanisms that promote and restrict eye formation to specific cell populations and tissues remains elusive.

The Notch, EGF Receptor (EGFR), Hedgehog (Hh) and TGFβ signaling pathways have been proposed as candidates for playing a permissive but required role in eye formation [14]–[17]. All three pathways play important roles in tissue and organ patterning, are expressed in tissues that support ectopic eye formation and are required for the proper functioning of the RD network in the eye primordium. These and other signaling cascades intersect the RD network at different points and in several cases appear to function reiteratively at many levels. For example, Notch signaling is required for *ey* activation [15], [16], [18]. The EGFR pathway also modulates *ey* expression but influences Eyes Absent (Eya) protein activity via phosphorylation by MAPK as well [15], [19]. Hh signaling is directly regulated by Sine Oculis (So) but then in turn activates *eya* expression thus forming a loop between the RD network and the Hh pathway [20]–[22]. In contrast, Wingless (Wg) signaling functions to establish the border between the eye and adjacent head tissue by repressing the expression of several RD network genes including *so*, *eya* and *dachshund* (*dac*) [23], [24].

The experimental evidence from the collection of papers describing ectopic eye formation suggests that, of the aforementioned signaling networks, the Hh and TGFβ pathways may be best candidates for being the requisite factor(s). This conclusion is been based, in part, on two lines of evidence. First, the position of induced ectopic eyes often intersects with the TGFβ and Hedgehog (Hh) expression domain [2], [4]–[8], [10]–[13], [17]. Second, co-expression of Ey and TGFβ or Hh in the wing expands the domain of ectopic eye formation [14], [17]. However, since the RD genes were expressed within just a handful of expression domains and most cells expressing TGFβ and Hh appear resistant to transformation into retinal tissue, the mechanism that may permit and restrict ectopic formation remains unclear.

In this study we have expressed eight RD genes either individually or in various combinations within 219 different expression domains. We confirm that only a discrete number of cell populations within the imaginal discs have the cellular plasticity to have their developmental fates altered. Increasing expression levels of individual RD genes or simultaneously expressing multiple RD genes does not expand the number of transformation “hot spots”. We also find that not all transformable cell populations lie within areas of high TGFβ and/or Hh signaling. It also appears that neither signaling cascade alone functions as the permissive factor for non-retinal to eye conversion. The transformation “hot spots” that we have identified appear to coincide with several previously defined transdetermination “weak spots”, suggesting that ectopic eye formation is less the result of one network overriding the orders of another as previously thought, but rather is the physical manifestation of redirecting cell populations of enormous cellular plasticity.

## Results

### Ectopic Eye Formation Is Restricted to Defined Cell Populations

**Figure 1:**
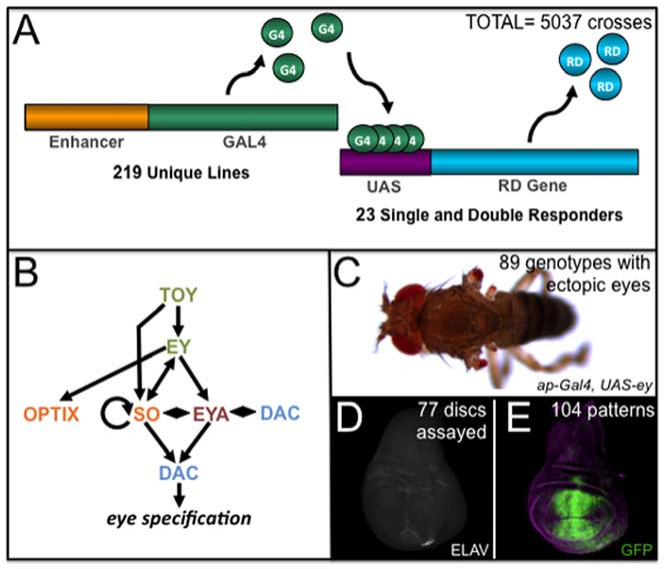
Screen for tissue competency to support eye development. (A) Schematic depiction of a GAL4 screen wherein 219-GAL4 driver lines were crossed to 8 single and 15 double responder lines for a total of 5037 genetic combinations. (B) A schematic representation of the retinal determination network containing only members that were expressed individually or in combination in this screen. Not show are the *eyegone* and *twin of eyegone* genes, which were also used in this study and that function to promote proliferation in the retina. (C) Example of an adult fly exhibiting ectopic eyes on the wings and halteres. (D) Visualization of GFP in the pGawB71B expression domain within the wing, which serves as an example of a broad expression pattern yielding an ectopic in a subset of cells. (E) Ectopic eye formation is observed by expression of ELAV, a pan-neuronal marker; note that region occupied by photoreceptors is less than that occupied by GFP.

In order to test the hypothesis that only select sub-populations of cells within the developing non-retinal epithelia are of sufficient cellular plasticity to support eye development we used the UAS/GAL4 system to conduct the following screen. Members of the RD network (alone or in combination; see Methods) were expressed in developing tissues under the control of 219 unique promoters (Fig. 1A,B). Of the 5037 different genotypes that were screened, we found ectopic compound eyes in the adults of 89 genotypes (Fig. 1C, Table 1). The location of the cell populations within third instar imaginal discs from which the ectopic eyes are derived were identified by the presence of two RD proteins Eyes Absent (Eya) and Dachshund (Dac) and the pan-neuronal protein, ELAV (Fig. 1D). Of the original promoter-GAL4 constructs we used a GFP reporter to determine the expression of the 25 promoters that are capable of expressing RD genes in the correct spatial and temporal patterns for promoting the formation of ectopic eyes (Fig. 1E). It should be noted that not all GAL4/UAS-RD gene combinations produced viable progeny. Of these, the most common lethal phase was embryogenesis. This is presumably due to the GAL4 line directing expression of RD genes during this developmental phase.

**Table 1:** List of GAL4 drivers, UAS-RD responders and Location of Ectopic Eyes.

Driver	UAS-Responder	Converted Tissue(s)
dpp-GAL4	toy	leg, wing
	ey	antenna, wing haltere, leg
	eya	antenna. leg
	so	antenna
	***ey eya***	***genitalia, labellum***
	***ey dac***	***genitalia, labellum***
	***so eya***	***ventral head***
	***so optix***	***ventral head***
pGawB71B-GAL4	toy	wing
	ey	leg
	***ey optix***	***wing***
ap-GAL4	ey	wing, haltere
	eya	wing
	***ey eya***	***leg***
	***toy eya***	***haltere***
bi-GAL4	toy	wing, leg
	ey	wing, haltere, leg
	eya	wing, haltere
pGawBcb16-GAL4	toy	leg
	ey	wing, leg
pGawBcb26-GAL4	eya	wing
pGawBcb32-GAL4	toy	leg
pGawB41-GAL4	toy	wing, leg
	ey	wing, leg
	eya	wing
	so	antenna
	***toy ey***	***antenna***
rn-GAL4	toy	wing, haltere
	ey	wing, haltere, leg
	optix	wing, haltere
	***toy ey***	***antenna***
	***toy dac***	***antenna***
	***ey eya***	***antenna***
	***ey dac***	***antenna***
pGawBcb49-GAL4	so	head, antenna
	optix	head, antenna
Ser-GAL4	toy	wing, haltere
	ey	wing
pGawBcb309-GAL4	optix	head, antenna
	***ey eya***	***leg***
	***eya dac***	***head***
pGawBc253-GAL4	optix	antenna
pGawBmj33a-GAL4	ey	wing
	ey eya	antenna leg
pGawB43-GAL4	eya	leg, genitalia
pGawBB7B-GAL4	dac	genitalia
GMR-GAL4	dac	head
pGawBdj847-GAL4	eya	head, wing, leg
sd-GAL4	ey	wing, haltere
	so	head
pGawB30A-GAL4	***ey optix***	***wing***
cad-GAL4	***ey so***	***wing, haltere, leg***
pGawB185Y-GAL4	***toy eya***	***leg***
pGawBc368-GAL4	***ey dac***	***leg***
wg-GAL4	***ey eya***	***leg***
pGawBcb50-GAL4	***ey dac***	***leg***
	***ey eya***	***leg***

Of the 5037 genotypes that were screened 57 contained ectopic eyes. Black text denotes the location of ectopic eyes that were observed when single RD genes were expressed. Bolded and italicized text denotes situations in which a combination of two RD genes resulted in the generation of ectopic eyes. Note that in these situations each single gene failed to induce ectopic eyes in the same location.

**Figure 2:**
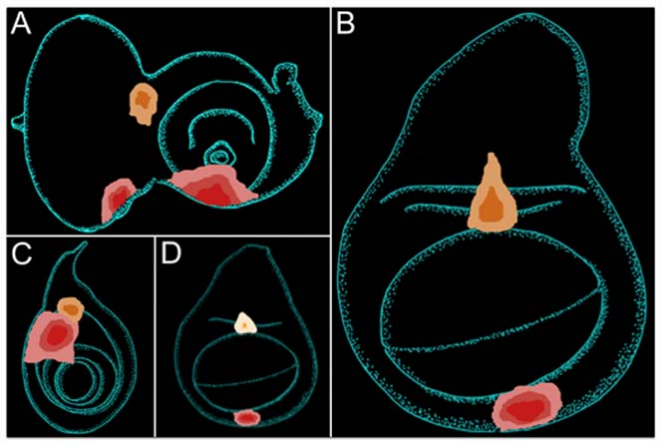
Schematic depicting cell populations capable of supporting retinal development. (A-D) Tissue transformation “hot spots” were determined by ELAV distribution. Red indicates primary hot spots, which are transformed at a higher frequency than secondary populations, which are marked in orange. The range of color shades within each hot spot denotes that the ectopic eyes range in size and frequency with the dark shade indicating that ectopic eyes more frequently transform a very limited population of cells. A = eye antennal disc, B = wing disc, C = leg disc, D = haltere. Anterior is to the right, dorsal is at the top.

The aforementioned hypothesis was confirmed as the transformation “hot spots”, which are depicted schematically in Figure 2, are the only topological areas within the eye, antennal, leg, wing and haltere imaginal discs where we were able to observe ELAV positive photoreceptor neurons. Interestingly, there are qualitative differences amongst the hot spots as certain cell populations (Fig. 2, red) are transformed at a much higher frequency than others (Fig. 2, orange). This difference is maintained even in situations in which promoter-GAL4 constructs, whose expression patterns cover both types of “hot spots”, are considered. We suggest that the more frequently transformed cell populations have greater developmental flexibility than the less frequently altered cells.

**Figure 3:**
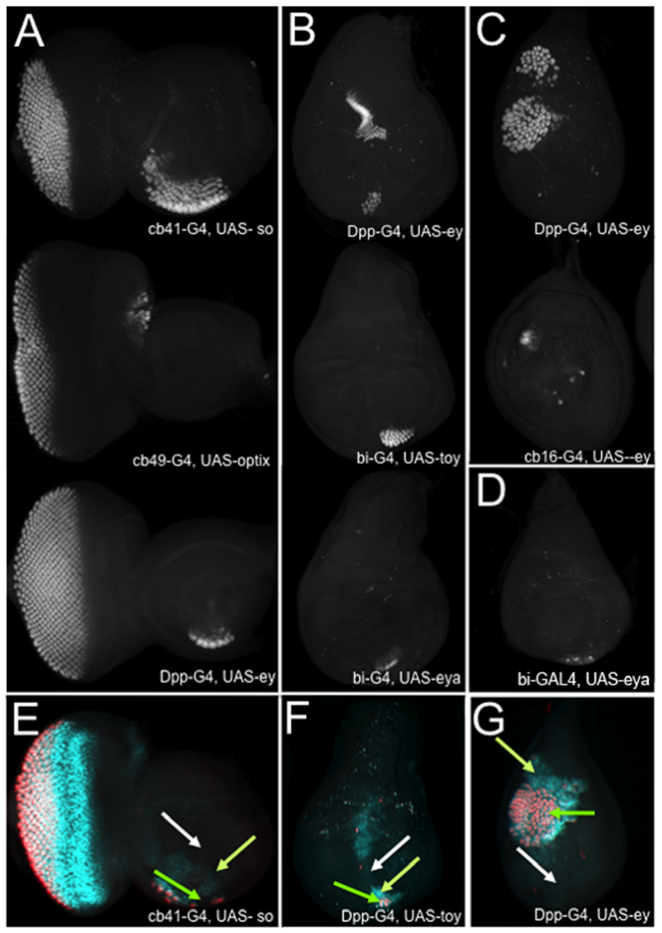
Ectopic eyes develop in a narrow range of cells within developing epithelial tissues. (A-D) Confocal images of third instar eye, antenna, leg, wing and haltere imaginal discs. These are representative images from the screen demonstrating that ectopic eye formation is limited to sub-populations of cells within each epithelium. Photoreceptor cells are marked by the presence of ELAV. A = eye antennal disc, B = wing disc, C = leg disc, D = haltere. Anterior is to the right, dorsal is at the top.

We also noted that the size of the ectopic eye and thus the number of cells that are transformed into photoreceptors is variable. The schematics in Figure 2 illustrate this observation in that the darker shades represent the size of the ectopic eyes that are seen most frequently while the lighter shades represent larger but rarer ectopic eyes. We suggest that this represents another example of the broad continuum of cellular plasticity that exists within developing imaginal tissues. Several examples of the topological range and positioning of the transformed cell populations are presented (Fig. 3A-D). In terms of the variable size of ectopic eyes within a single “hot spot”, it is unclear if all ectopic eyes start at the same point and grow to varying sizes due to differential advancement of morphogenetic furrows or whether varying sizes of cell populations are initially transformed (this model does not require a moving furrow). We tend to favor the second model, as we often see no evidence of an advancing furrow (assayed using *dac* expression as a marker). We also do not observe the normal pattern and progression of photoreceptor neuron differentiation within the ectopic eyes that is seen in the normal eye. Instead, in most cases during development, ommatidial clusters at the edges of the ectopic eye appear as mature as those located in more central regions.

### TGFβ and Hh Signaling Are neither Absolutely Required nor Sufficient for Ectopic Eye Formation

If only specialized cell populations are competent to support eye formation it raises the issue of what is the cellular and molecular environment underlying this capacity. Prior reports have suggested that normal and ectopic eye formation requires the presence of high TGFβ and Hh signaling [14], [17]. Indeed, prior to our study, all ectopic eyes were found to be located within these expression zones. Furthermore, co-expression of *ey* with either *decapentaplegic (dpp)* or *hh* induced ectopic eyes outside of the A/P axis and posterior compartment. One implication of these results is that any and all cell populations that can support ectopic eye formation should lie within high Dpp and/or Hh expression zones. While most transformation “hot spots” are found within the Dpp expression domain, two cell populations, one within the eye (Fig. 2A, orange) and another within the leg (Fig. 2C, orange), lie outside of this geographic area. In the case of the eye this hot spot also lies outside the Hh zone. A second implication of these previous models is that all cells within the Dpp and Hh expression zones are competent to adopt a retinal fate. We find that is not the case by observing that in each imaginal disc only a subset of cells along the A/P axis and posterior compartment, which express Dpp and Hh respectively [25], have the degree of cellular plasticity to adopt a retinal fate (Fig. 3E-G, green arrow). We find this to be significant as it suggests that the requirements for eye formation are likely to be very extensive, complex and possibly tissue specific.

### Expression of RD Genes Reveals Cryptic Degrees of Developmental Plasticity

We were interested in observing the temporal process of ectopic eye formation. In the normal eye, the RD network is expressed at one time or another within all cells that constitute the eye primordium [1]. The initiation of the morphogenetic furrow at the posterior margin and its passage across the epithelium converts this entire undifferentiated field into the adult retina [26], [27]. In all instances of ectopic eye formation we observe that the expression of RD genes (single members or combinations) is only sufficient to activate the rest of the RD network in a subset of cells within the chosen GAL4 expression domain. This zone of RD network expression, without exception, is larger than and encompasses the cells that will eventually adopt a retinal fate (Fig. 3E-G). Our findings suggest that there are several levels of tissue competency. Within a tissue many cell populations are completely refractory to any effects of RD gene expression (Fig. 3E-G, white arrow). Other cell populations display an intermediate level of plasticity by activating the expression of the RD network but remain incapable of supporting photoreceptor neuron fates (Fig. 3E-G, light green arrow). And finally, cell populations with the highest cellular plasticity completely adopt a retinal fate (Fig. 3E-G, green arrow). It is unclear if all of these cells are also capable of adopting other terminal fates or does that ability constitute an even smaller cell population.

### Increased Gene Dosage Does Not Alter Location and Size of Transformation Hot Spots

**Figure 4:**
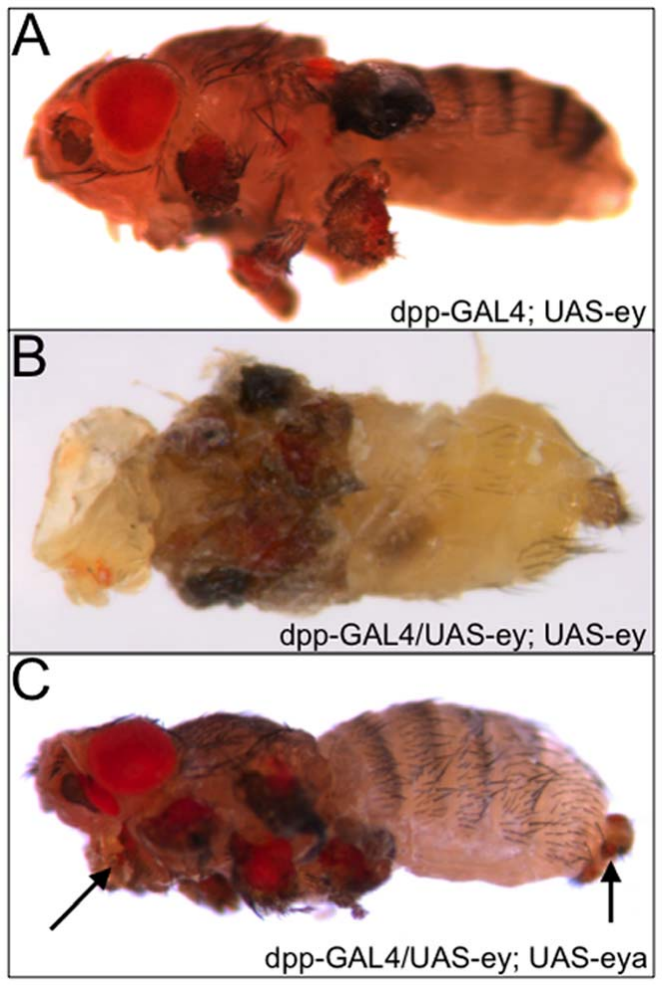
Increases in RD gene dosage are insufficient to specify additional ectopic retinal tissue. (A-C) Light microscope images of adult flies containing ectopic eyes in non-retinal tissues. Genotype is noted at the lower right hand corner of each panel. Note that in panel C, the UAS-ey, UAS-eya combination can induce new ectopic eyes in the genitals (arrow). Compare this with the expression of two copies of UAS-ey (panel B), a manipulation that fails to induce eye formation in the genitals. Anterior is to the left.

We set out to determine if the limits in cellular plasticity that we observe in our ectopic eye assay is a reflection of either not expressing individual RD genes at a high enough level or by not expressing the correct combination of RD network branches. To answer the first question, we drove two copies of *ey*, which is the most potent inducer of ectopic eye formation [2], [28] and observe no increase in the size of ectopic eyes, no change in the tissue distribution of the eyes and no variation in the location of the transformation hot spots (Fig. 4A-B). To answer the second question we made and expressed fifteen different double UAS-RD gene combinations in 219 unique expression domains. Of the 3285 double UAS-responder genotypes screen, we observed only a few rare instances (0.7%) in which expression of an RD combination gave an ectopic eye within a tissue that was not seen with either single RD gene and the same GAL4 driver (Table 1). One example of this occurs when both *ey* and *eya* are co-expressed within the genitals. While the individual genes fail to induce ectopic eyes here, the combination is sufficient to induce eye formation (Fig. 4C, arrow). We did identify another tissue that is typically refractory to retinal development that we were able to coax towards an eye fate, the labellum (Fig. 4C, arrow). This is a novel location that we were able to transform with UAS-ey, UAS-eya double responders. However, in spite of these rare cases, we did not observe a change in the location of the cellular transformations in the epithelial tissues shown in Figure 2. In these tissues, only cells that we have identified as transformation hot spots are capable of supporting eye development.

### Timing of Normal and Ectopic Eye Formation Is Asynchronous

**Figure 5:**
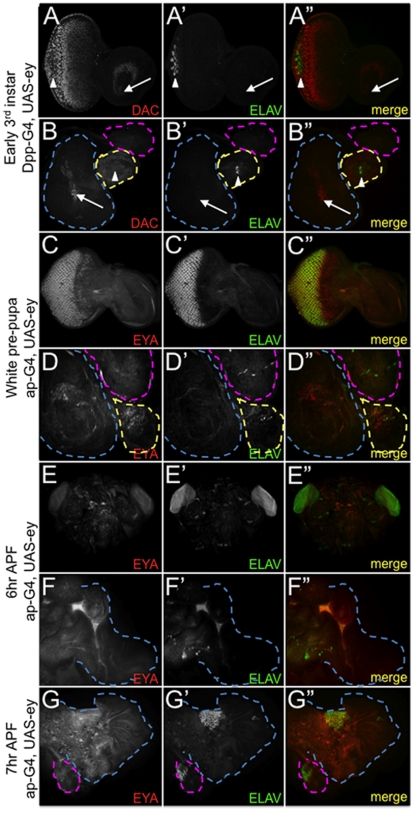
Onset of eye development in non-retinal tissues is asynchronous to the normal eye. (A-G) Confocal images of third instar (A,B), white prepupae (C,D) and early pupal (E-G) imaginal discs/ pupal tissues. (A,B) At the onset of retinal specification in the normal eye (A, arrowhead), retinal specification has not yet begun in either antennal or leg ectopic eyes. Note the ectopic dac expression in the antenna and leg lacks ELAV (A,B arrow). The ELAV positive cells within the leg disc (B, arrowhead) are normal non-retinal neurons. (C-G) Normal eye specification is well underway (C,E) but photoreceptors in the wing and haltere have yet to be specified. For each stage the eye antennal, leg, wing and haltere discs are from the same individual. Developmental stage and genotype are listed at left.

In several instances, while ectopic eyes were observed in adult tissues, we were unable to detect ELAV positive photoreceptor neurons within the non-retinal third larval instar tissues even though development in the normal eye proceeded normally. This led us to test the hypothesis that the timing for ectopic eye formation is asynchronous to that of the normal eye. We examined normal and ectopic eye development in dpp-GAL4/UAS-ey animals, a genotype that generates ectopic eyes within the antenna, legs, wings and halteres [2] and ap-GAL4/UAS-ey where eyes are generated in the wing and haltere [28]. At the early third instar larval stage, when the normal eye contains 1–5 rows of ommatidia (Fig. 5A, arrowhead), ectopic photoreceptor neurons in dpp-GAL4/UAS-ey animals were not present in any of the expected non-retinal epithelium despite the activation of *dac* and *eya* expression (Fig. 5A-B). Similarly, in ap-GAL4/UAS-ey animals we did not observe the formation of ectopic eyes in either white pre-pupa or 6hr pupa (Fig. 5C-F). With this genotype we see the first signs of ectopic ELAV positive neurons being specified in 7hr pupa (Fig. 5G). Since the dpp-GAL4 and ap-GAL4 drivers are activated early in development (first instar for dpp-GAL4 and embryogenesis for ap-GAL4) the differences that we see in ectopic eye initiation (as compared to the normal eye) are likely attributable to differences in developmental timing mechanisms.

## Discussion

While a series of reports have documented the induction of ectopic eyes by members of the RD network [2], [4]–[12] little comment, save two exceptions [14], [17], has focused specifically on the observed spatial constraints surrounding the transformation of non-retinal epithelia into eye tissue. Such restrictions are often attributed to limited choices of temporally and spatially relevant enhancer elements (which are used to drive expression of RD genes), inadequate expression levels of RD genes (use of single gene copies) and the activation of only sub-circuits of the network (due to the expression of individual RD genes). Here we present evidence for an alternative hypothesis: that there are a discrete number of cell populations that are of sufficient developmental plasticity to have their primary developmental instructions superseded and thereby allowing for the adoption of a retinal fate. This is based on a screen for ectopic eyes in 5037 unique over-expression genotypes in which all ectopic eyes in the eye, antennal, leg, wing and haltere fields were found within nine discrete cell populations.

It has been argued that the TGFβ and Hh signaling pathways are essential and required elements for the induction of ectopic eyes. This model is based on observations that many ectopic eyes appear to be positioned along the Dpp and Hh expression domains and that co-expression of Eyeless with Dpp or Hh increased the range where ectopic eyes are located [14], [17]. However, our findings suggest that these signaling pathways, while important for eye formation, are not sufficient for the promotion of ectopic eye development as most cells within the Dpp and Hh expression domains of multiple tissues are refractory to the forced expression of individual or combinations of RD genes. We also observe that of the nine identified cell populations that can support eye development; two are not located within the Dpp expression domain. Of these two regions, one is also positioned away from the Hh expression zone. Together, these results suggest that the mechanisms that underlie the permissiveness with which certain cell populations can adopt different fates is more complex than previously envisaged. Other mechanisms might include the Polycomb Group (PcG) and trithorax Group (trxG) chromatin regulatory proteins as well as other signaling pathways such as the Wnt cascade.

Three of our transformation hot spots (1 each within the eye, antenna and leg) lie tantalizingly close to, if not within, the transdetermination weak points that have been previously reported [29]–[31]. In *Drosophila*, while imaginal discs can retain their primary fate during extended periods of *in vivo* culture, a few examples were observed in which the cells would adjust to another disc fate, a process termed transdetermination [32]. The Wnt signaling pathway has been implicated to play a key role in this process as high levels of Wingless expression can induce transdetermination within select subpopulations of cells [29]–[31]. A recent study has also implicated members of the PcG and trxG complexes in transdetermination events [33]–[34]. Members of the eye specification network (*eyes absent* and *eyegone*) can repress Wnt expression [34] thereby eliminating one negative regulator or eye development [23], [24] within the transdetermination weak points. But as the PcG and trxG members are key players in transdetermination, cells within the weak point may still retain some of their developmental plasticity and can adopt a retinal fate. We propose that in at least three instances, the induction of ectopic eye formation has occurred within the population of cells that are capable of transdetermining and thus represent the cells with the highest developmental plasticity. We also suggest that the other six cell populations identified in our study may represent additional transdetermination weak points that were not detected in prior studies.

The formation of ectopic eyes in several ways deviates from the pattern of development seen within the normal eye field. First, the onset of eye development appears to be regulated by different molecular clocks than the normal eye and the particular timing is dependent upon individual tissues. Second, unlike the normal eye, once an ectopic eye is initiated it is incapable of transforming and spreading across the entire epithelium. Rather, the ectopic eye appears to respect a border whose molecular identity is yet to be determined. And finally, growth of the ectopic eye appears to not always make use of a morphogenetic furrow suggesting that in some cases an entire patch of tissue simultaneously adopts a retinal rate, much like the area that gives rise to the first 1–4 ommatidial rows within the normal eye. It will be interesting to (1) elucidate the molecular factors that distinguish ectopic eye from normal eye development; (2) determine if all of our transformation hot spots are indeed transdetermination weak points; and (3) if other cell populations can be induced into this state. The answers to these questions may go a way to understanding why *Drosophila* imaginal discs have such cell populations in the first place.

## Methods

### Fly Stocks

Expression of retinal determination genes via the following GAL4 drivers resulted in ectopic eye formation: dpp-Gal4, pGawB71B-Gal4, ap-Gal4; bi-Gal4, pGawBcb16-Gal4, pGawBcb26-Gal4, pGawBcb32-Gal4, pGawBcb41-Gal4, rn-Gal4, pGawBcb49-Gal4, Ser-Gal4, pGawBc309-Gal4, pGawBc253-Gal4, pGawBmj33a-Gal4, pGawB43-Gal4, pGawB7B-Gal4, GMR-Gal4, pGawBdj847-Gal4, sd-Gal4, pGawB30A-Gal4, cad-Gal4, pGawB185Y-Gal4, pGawBc368-Gal4, p(Gal4-wg.M), pGawBcb50-Gal4. A complete list of GAL4 drivers used in this study is available on request.

The following responder lines were used to express individual or combinations of retinal determination genes: UAS-ey (gift of Georg Halder), UAS-ey, UAS-toy, UAS-optix (gift of Walter Gehring), UAS-toy; UAS-eya (gift of Nancy Bonini); UAS-so; UAS-dac (gift of Graeme Mardon); UAS-eyg; UAS-toe. A UAS-GFP (Bloomington Stock Center) was used to determine the expression domain of GAL4 drivers. The following double RD gene combinations were generated and used in this study: UAS-ey, UAS-eya; UAS-ey, UAS-dac; UAS-toy, UAS-optix; UAS-toy, UAS-so; UAS-toy, UAS-eya; UAS-toy, UAS-dac; UAS-optix, UAS-so; UAS-optix, UAS-eya; UAS-so, UAS-eya; UAS-so, UAS-dac; UAS-eya, UAS-dac.

### Antibodies, Immunohistochemistry and Light Microscopy

The following antibodies and reagents were used in this study: rat anti-ELAV (1∶10, Developmental Studies Hybridoma Bank), goat anti-rat FITC (1∶20, Jackson Laboratories), phalloidin-TRITC (1∶100 Molecular Probes). 3^rd^ instar and pupal stage imaginal discs were dissected, fixed and prepared for confocal microscopy as described in [35]. Adult flies containing ectopic eyes were prepared and photographed as described in [28]. The age of pupae was determined by collecting white prepupa and aging the animals at 25°C.
